# Determinants of maternal knowledge of neonatal danger signs among postnatal mothers visiting neonatal intensive care unit, north Central Ethiopia, 2019: a cross-sectional study

**DOI:** 10.1186/s12884-020-02896-x

**Published:** 2020-04-15

**Authors:** Wubet Alebachew Bayih, Biniam Minuye Birhan, Abebaw Yeshambel, Molla Asfaw

**Affiliations:** Department of Nursing, College of Health Sciences, Debre Tabor University, P.O.BOX 272 Debre Tabor, Ethiopia

**Keywords:** Knowledge, Neonatal danger sign, Debre Tabor

## Abstract

**Background:**

Sick neonates can be early readmitted if and only if their mothers have good knowledge of the key neonatal danger signs at first discharge. Thus, it was aimed to assess the level and determinants of maternal knowledge on these signs at first discharge from NICU.

**Methods:**

A hospital based cross sectional study design was employed at Debre Tabor General Hospital, South Gondar Zone. A sample of 363 participants was included to the study from September 2018 to February 2019 through systematic selection of every other eligible mother baby pair. Data were collected through face to face interview at time of discharge from NICU. Knowledge score of neonatal danger signs was computed by adding the total number of correct spontaneous responses to 9 key danger signs with a minimum score of 0 and maximum of 9 [0 when a mother named none of the key danger signs and 9 when the mother named all the signs]. Mothers who scored ≥3 points were considered to have good knowledge whereas those scoring less than 3 points had poor knowledge.

**Results:**

224(61.70%) mothers had good knowledge of neonatal danger signs at discharge from NICU. Secondary and above level of education [AOR = 4.62], receiving danger sign information during stay at NICU [AOR = 3.64], four and above antenatal visits [AOR = 3.04], well preparedness of birth [AOR = 13.70], institutional delivery [AOR = 6.46] and good knowledge of essential newborn care [AOR = 4.41] were significant factors.

**Conclusions:**

At discharge time, maternal knowledge of neonatal danger signs wasn’t comparable to their exposure of NICU environment. Therefore, danger sign education should be routinely given during maternal stay at NICU. Moreover, existing efforts should be enhanced to improve number of antenatal visits, institutional delivery rate and postnatal services along the continuum of maternal and child health care in South Gondar Zone.

## Background

Key neonatal danger signs are severe non-specific manifestations of serious neonatal illness that must be early recognized and promptly managed [1–4]. These signs are: 1) fever 2) not being able to breastfeed 3) difficulty of breathing /severe chest in-drawing 4) lethargy/ unconsciousness 5) Coldness /hypothermia 6) Yellow palms and soles 7) Pus discharges from the umbilicus 8) convulsion and 9) redness/ eye discharge [1, 3–5]. Different studies in Ethiopia [6–14] and other countries [15, 16] emphasize the essence of good maternal knowledge on these signs to reduce neonatal mortality. Moreover, these studies have benchmarked the cut off score for good maternal knowledge of danger signs, which is mentioning at least 3 of the overall 9 key danger signs [6–10, 12–14]. This is so because knowing a minimum of 3 key danger signs help to give more specific and sensitive prediction of the need for hospitalizing neonates during illness [3–5]. Taking all the 9 danger signs into consideration, this study also bench marked the same cut off point to score maternal knowledge of danger signs at first discharge from NICU. Mothers were purposively selected from NICU because neonates surviving from their first admission have the likelihood of getting sick again. These neonates can be early readmitted if and only if their mothers have good knowledge of the key neonatal danger signs at first discharge from NICU [1, 3, 5].

Compared to other children, neonates face the highest risk of dying at a global rate of 19 deaths per 1000 live births. Globally, neonatal deaths accounted 46% of all under-five deaths [17, 18]. Among these deaths 38 and 39% occurred in Southern Asia and sub-Saharan Africa respectively. In sub-Saharan Africa, about 1 child from 36 children dies in the first month of life, which is very high compared to developed countries (i.e.1 in 333) [19]. Ethiopia is among the six neonatal mortality burden countries in the world [18]. The high neonatal mortality rate across these countries is corresponding to mothers’ poor knowledge of neonatal danger signs. For example, in Ethiopia (71%), Uganda (85%), Nigeria (70%) and India (38%) of the mothers had poor knowledge of neonatal danger signs [15, 16, 20, 21]. From the different regions in Ethiopia, Amhara region (a region of the study area) contributes the highest neonatal mortality burden (47%) of the nation [22].

Although Ethiopia has taken a great initiative to empower the health professionals for improving neonatal health services at the grass root level, maternal knowledge level about neonatal danger signs was found to be low. This could in turn accelerate the death of neonates [6–14]. From prior studies, some of the main determinants of poor maternal knowledge about neonatal danger signs were rural residence, maternal occupation, home delivery, lack of antenatal and postnatal care follow ups, no spousal accompaniment to antenatal and postnatal care follow ups, low level educational status, primiparity, poor knowledge of essential newborn care, poor access to mass media, lack of birth preparation and complication readiness plan [5–18, 23, 24].

According to 2016 Ethiopian Demographic Health Survey (EDHS), Ethiopian Neonatal Mortality Rate (NMR) is 29 per 1000 live births [22]. According to target 3.2 of the health goal of the Sustainable Development Goal (SDG), Ethiopia has promised to reduce NMR from 29 per 1000 live births to 12 per 1000 live births by 2030 by ending preventable neonatal deaths [23]. Achievement of this target requires maternal early recognition and immediate health care seeking behavior of key neonatal danger signs [3, 8]. However, there were no studies conducted so far about the level and determinants of maternal knowledge of neonatal danger signs at first discharge from NICU. Even prior studies of similar topics conducted at settings other than NICU showed variation among their findings [6–14]. Therefore, this study was aimed to reach findings that aid in strategizing programmatic interventions which enable mothers to acquire good knowledge and immediate health care seeking behavior of key neonatal danger signs during their stay at NICU.

### Conceptual framework

**Fig. 1:**
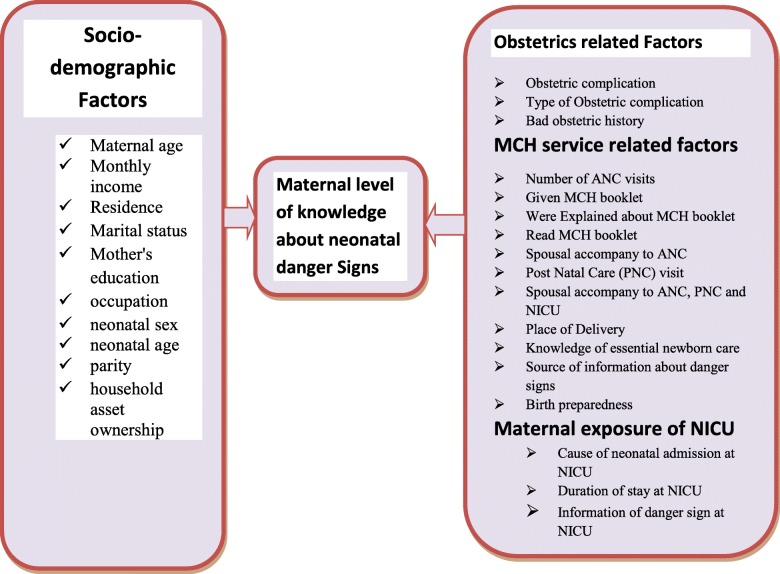
Conceptual frame work for the determinants of maternal knowledge of neonatal danger signs presented to NICU of Debre Tabor General Hospital, developed after reviewing available literatures regarding the problem, 2019

After reviewing different literatures, conceptual framework of the study was developed based on the most influential and widely cited framework proposed by Mosley and Chen in 1984 for the study of child survival in developing countries. The basic idea of Mosley-Chen framework is that all social, economic, cultural and health system related variables impact child survival through a set of proximate determinants. The framework has been improved and used in a range of longitudinal and cross-sectional studies of child survival by incorporating these variables and designed to integrate research methods from demography and epidemiology. Based on the framework, this study conceptualized determinants of maternal knowledge of neonatal danger signs to be resulted from the interaction of various factors labeled as socio-demographic, maternal and child health service related, obstetrics history related and maternal exposure of NICU as shown by Fig. 1 [24].

## Methods

### Study setting and period

The study was conducted at Debre Tabor General Hospital (DTGH), which is 666kms North East of Addis Ababa, capital of Ethiopia. It is the only governmental hospital in Debre Tabor town established in 1930. The hospital serves to a population of nearly 2.7million and linked to 7 district hospitals. As per the hospital’s report of 2017, there were 20,000 women of reproductive age group getting reproductive health service from the hospital in the year. Moreover, report of the same year revealed that the average number of deliveries served by the hospital annually was 3719 (123.9%), beyond its plan, 3000. Neonatal Intensive Care Unit (NICU) of the hospital hosted approximately 1159 admissions per year, as per the hospital’s report of 2017. Most of the admissions had danger signs attributed to mainly prematurity, perinatal asphyxia, neonatal sepsis and congenital malformation. Nurse to neonate ratio of the hospital in NICU was 1 to 5. The study was conducted from September 2018 to February 2019 (Debre Tabor General Hospital Annual Report [unpublished]).

### Study design and participant characteristics

Institution based cross sectional study design was employed. All mother- neonate pairs fulfilling the inclusion criteria and visiting NICU during the study were eligible. However, 2 abandoned neonates (those neonates left in NICU without mothers) were excluded as there was no other source of subjective data for these neonates. Moreover, 4 mothers with the trouble of talking/listening and 2 mothers having psychiatric disorders were excluded as they were not mentally and physically capable of being interviewed. Three non volunteer mothers were also excluded. Besides, 8 readmitted mother-neonate pairs were excluded because the study was aimed at assessing maternal knowledge of neonatal danger signs at first discharge from NICU.

### Sample size determination and sampling procedure

By using single population proportion formula and considering confidence level /Z/ of 95%, marginal error of 5%, a reasonable estimate for the proportion of good maternal Knowledge of neonatal danger signs (*P* = 0.31) from a previous study done at Southern Ethiopia, 2017 [19] and adding a none response rate of 10%, a total sample of 363 mother-baby pairs was obtained. By using systematic sampling, all the selected women (every other eligible mother) agreed to participate in the study thereby making a response rate of 100%.

### Measurement and data collection procedure

Data were collected by four trained personnel who had Bachlor of Science (BSc) degree in neonatal nursing through face to face interview using a pretested and structured questionnaire. Before leaving NICU, an exit interview was made for every other eligible mother. The questionnaire contained factors related to maternal socio demography, MCH service, birth preparation and NICU related factors. Moreover, 9 WHO adopted key neonatal danger signs were considered to measure the level of maternal knowledge on these signs [3, 18]. These signs were: 1) fever 2) being unable to breastfeed 3) difficulty of breathing 4) lethargy/unconsciousness 5) Coldness /hypothermia 6) Yellow palms and soles 7) Pus discharge from the umbilicus 8) convulsion 9) redness/ eye discharge.

### Operational definition

Knowledge score was computed by adding the total number of correct spontaneous responses to 9 items with a minimum score of 0 and maximum of 9 [0 when a mother mentioned none of the key danger signs and 9 when the mother mentioned all the danger signs]. Spontaneous response is respondents’ naming of neonatal danger signs without giving option of the respective signs. Accordingly, two categories were developed for knowledge of neonatal danger signs [Good and poor categories]. Women who mentioned at least three neonatal danger signs were considered to have good knowledge whereas those who mentioned less than three of the danger signs were labeled to have poor knowledge as stated by several studies [6–10, 12–14].

Moreover, good knowledge of essential newborn care was regarded when mothers responded greater than 50% of knowledge related questions correctly whereas it was poor if responded less than or equal to 50% of the knowledge related questions [25].

### Data quality control methods

Amharic version anonymous questionnaire was used for data collection. The tool was adapted from the safe motherhood questionnaire developed by the non-profit organization JHPIEGO [26], studies in Ethiopia [6–14], other African studies [15–17, 27] and India [20, 28].

Four days of training (3 days of theoretical training and 1 day of practical training) was first provided for data collectors and supervisors about pretesting and the process of data collection. Before the actual data collection, pretest was done using 18 eligible mother-baby pairs (5% of sample size) at Nefas Mewucha District Hospital, which is nearby to the study hospital, just to evaluate the clarity of questions, validity of the instrument and reaction of the respondents to the questions. During data collection, data collectors were closely monitored and guided by two MSc neonatal nurse supervisors for complete and appropriate collection of the data. Reporting of the collected data to the principal investigator was made on a daily basis. Furthermore, the collected data were double entered into Epidata version 4.2 by two data clerks for validation purpose.

### Data processing and analysis

The collected data were coded, cleaned, edited and entered into epidata version 4.2 after which it was exported to SPSS version 23 software for further analysis. Frequencies, proportion, summary statistics and cross tabulation were used to describe the study population in relation to relevant variables and presented in tables. The assumptions for binary logistic regression model were first checked and then bivariable analysis was carried out to identify as many candidate variables as possible (*p* < 0.25) for multivariable analysis. Then multivariable logistic regression analysis was performed using those candidate variables to investigate statistically significant predictors of maternal knowledge of neonatal danger signs by adjusting for possible confounders. Finally, variables whose *p* value less than 0.05 (*p* < 0.05) from multivariable logistic regression were declared as statistically significant using adjusted odds ratio of 95% CI. Multi-collinearity between the study variables was diagnosed using standard error and correlation matrix. Hoshmer-Lemeshow statistic and Omnibus tests were also performed to test for model fitness.

### Ethical consideration

Ethical clearance was obtained from ethical review committee of Debre Tabor University. Following the approval, official letter of co-operation was given to the hospital manager. Because most of the mothers at NICU were unable to read and write, the usual verbal voluntary consent was obtained from each mother after giving explanation of the study. All the participants were above 16 years old and hence parental consent wasn’t required. As the study was conducted through face to face interview, the individual participants were not exposed to any physical harm. Moreover, the mothers were told that the information they gave to be treated with complete confidentiality. There was an already organized counseling team in NICU whose primary role was giving psychological advice and reassurance of emotionally distressed mothers. Thus, the authors used to link emotionally distressed mothers to this counseling team so that mothers could get relieved of their stress. After every interview, each mother was taught of all the neonatal danger signs and provided with a template that contained the danger signs in local language. Moreover, they were informed of practicing immediate health care seeking to nearby clinic at times of identifying the signs.

## Results

### Socio-demographic factors

The mean maternal age was 29.4 (SD = ±5.4) years. More than two-third (67.8%) of the mothers were rural residents. Moreover, 93.4% of the mothers were married, 65.0% housewives and 37.5% unable to read and write. About three-fifth (59.8%) of the mothers had an average monthly family income of below poverty line. More than three fourth of the mothers (76.3%) had low household asset ownership as shown in Table 1.

**Table 1:** Socio-demographic and reproductive characteristics among postnatal mothers visiting Neonatal Intensive Care Unit of Debre Tabor General Hospital, Debre Tabor town, North Central EthiopiLa, 2019

Factor (*n* = 363)	Percentage
Maternal age (years)	
16–25	29.5
25–34	58.7
> 34	11.8
Parity	
1	30.0
2–4	43.0
≥ 5	27.0
Residence	
Urban	36.4
Rural	63.6
Marital status	
Married	93.4
Other^a^	6.5
Educational level	
Unable to read and write	37.5
Primary education	33.9
Secondary and above	28.6
Occupation	
Merchant	9.6
House wife	65.0
Civil servant	20.1
Other^b^	5.2
Average monthly income ($ USA) [28.94 ETB =1 USD]	
< 37.5	59.8
≥ 37.5	40.2
Household asset ownership	
Low (0–1 items^c1^)	76.3
Moderate (≥2 items^c1^)	23.7

**Other**^a^ refers to divorced and separated

**Other**^b^ refers to student, laborer and private worker (hair dresser, machine operators, typewriters)

^c1^ Radio, television set, mobile phone, bicycle, motorcycle and car/truck

### Maternal and child health service related factors

Three hundred eight (84.8%) of the mothers had at least one antenatal care visit and 69.5% of them were accompanied by their spouse to ANC clinic. One hundred five (34.1%) mothers attended four or more times of ANC visit. During antenatal care visit, about 79.9% of the mothers were given MCH booklet. However, only 27.6% of them were given information of neonatal danger signs as depicted in Table 2.

**Table 2:** Maternal and child health service related factors among postnatal mothers visiting Neonatal Intensive Care Unit of Debre Tabor General Hospital, Debre Tabor town, North Central Ethiopia, 2019

Factor	Percentage
ANC follow up (*n* = 363)	
Yes	84.8
No	15.2
Number of ANC visit (*n* = 308)	
< 4 times	65.9
≥ 4 times	34.1
Accompanied by spouse to ANC (*n* = 308)	
Yes	69.5
No	30.5
MCH booklet (*n* = 308)	
Were given during ANC	79.9
Were explained on its contents	40.3
Received information about neonatal danger signs	27.6
Read all the instructions in the booklet during ANC	32.8
Delivery place (*n* = 363)	
Health institution^a^	62.5
Home	37.5

^a^ Health centers and hospital

### Birth preparedness

From the overall mothers, it was found that only192 (52.9%) of them were prepared of their birth with complication readiness plan as shown in Table 3.

**Table 3:** Birth preparedness for the index neonate among postnatal mothers visiting Neonatal Intensive Care Unit of Debre Tabor General Hospital, North Central Ethiopia, 2019

Factor	Percentage
Birth preparedness (*n* = 363)	
Saved money	71.4
Identified transportation	82.4
Bought childbirth materials	20.7
Identified skilled birth attendant	28.9
Well prepared^a^	52.9

^a^A mother was considered ‘well prepared’ of her birth if and only if she addressed at least three of the above numbered list of criteria

### Obstetrics related factors

Twenty nine (8.0%) of the mothers had history of bad obstetrics of whom the majority (62.1%) suffered from neonatal death in their prior pregnancies. Moreover, about 21.8% of the mothers reported having different obstetric complications during pregnancy of their index neonate. From these complications, preeclampsia/eclampsia contributed the highest percentage (59.5%) as displayed in Table 4.

**Table 4:** Obstetric factors among postnatal mothers visiting Neonatal Intensive Care Unit of Debre Tabor General Hospital, Debre Tabor town, North Central Ethiopia, 2019

Factor	Percentage
History of bad obstetrics (*n* = 363)	
Yes	8.0
No	92.0
If yes, which one (*n* = 29)^a^	
Neonatal death	62.1
Abortion	37.9
IUFD	6.9
Still birth	13.8
Obstetric complication during pregnancy of the index neonate (*n* = 363)	
Yes	21.8
No	78.2
Type of obstetric complication^a^(*n* = 79)	
Pre eclampsia/eclampsia	59.5
Abnormal labor^b^	29.1
PROM	11.4
APH	8.9
PPH	2.5
Other (DVT)	1.3

^a^multiple responses were obtained

^b^Obstructed labor, precipitated labor, prolonged labor, induced and/augmented labor

*DVT* Deep Vein Thrombosis

### Postnatal care (PNC) visit

Two hundred thirty seven (65.3%) of the mothers were given the first postnatal care. However, it was only 7.7% of them that received the second PNC despite the national schedule. Moreover, mothers who were accompanied by their spouse to PNC accounted for only 25.1% as shown in Table 5.

**Table 5:** History of post natal care among postnatal mothers visiting Neonatal Intensive Care Unit of Debre Tabor General Hospital, Debre Tabor town, North Central Ethiopia, 2019 (*n* = 363)

Factor	Percentage
PNC visit	
Within 24 h of birth	65.3
(48 – 7) days after birth	7.7
Accompanied by spouse to PNC	
Yes	25.1
No	74.9

*NB* the third PNC visit wasn’t considered as neonates are younger than this visit period

### Maternal exposure of neonatal intensive care unit (NICU)

Regarding causes of neonatal admission, prematurity attributed the highest (38.3%) proportion followed by perinatal asphyxia (25.6%). Most of the mothers (80.7%) stayed at NICU for utmost 14 days. Despite this duration of stay, it was only about 63.4% of the overall mothers who received information of neonatal danger signs during their stay. Moreover, 64.7% of the mothers weren’t accompanied by their spouses during their stay at NICU as shown in Table 6.

**Table 6:** Maternal NICU exposure related factors among postnatal mothers visiting Neonatal Intensive Care Unit of Debre Tabor General Hospital, Debre Tabor town, North Central Ethiopia, 2019 **(*n*** **= 363)**

Factor	Percentage
Cause of admission to NICU	
Prematurity	38.3
Perinatal asphyxia	25.6
Neonatal sepsis	18.5
Congenital malformation	9.6
Other*	11.3
Duration of stay at NICU	
< 14 days	80.7
≥ 14 days	19.3
Received information about danger signs during stay at NICU	
Yes	63.4
No	36.6
Accompanied by spouse during NICU stay	
Yes	35.3
No	64.7

Other* refers to jaundice, birth trauma

### Maternal knowledge of essential newborn care (ENC)

Mothers were asked of questions regarding the essential care of their newborns and it was found that 25.1% of them responded the use of new blade to cut the cord. Concerning the material for cord ligature, 48.8% of the mothers claimed the use of string/thread. To support the cord stump, 13.5% of the overall respondent mothers acknowledged the application of dressing. Different materials were also stated to be applied to the stump of which vaseline was most mentioned (11.3%). Moreover, about 27% of the mothers responded bathing of their newborns before 24 h of age and 66.1% explained the initiation of breastfeeding within one hour of delivery (Table 7).

**Table 7:** Maternal knowledge of essential newborn care among postnatal mothers visiting Neonatal Intensive Care Unit of Debre Tabor General Hospital, Debre Tabor town, North Central Ethiopia, 2019 (*n* = 363)

Factor	Percentage
Material for cord cutting	
New blade	25.1
New scissor	74.9
Material for cord ligation	
String or thread	48.8
Cord tie	32.8
Cord clamp	10.7
Don’t know	7.7
Material application on cord stump	
Nothing applied	79.6
Butter	8.5
Vaseline	11.3
Other (animal dung)	0.6
Cord stump support	
Without dressing	86.5
With dressing /cover	13.5
Neonatal bathing	
After 24 hrs of delivery	72.7
Before 24 hrs of delivery	27.3
Initiation time of breastfeeding	
Within 1 hr of delivery	66.1
After 1 hr of delivery	20.9
After placenta was removed	11.0
Don’t know	1.9

Based on the median score for knowledge questions of ENC, 57.3% of the mothers had good knowledge of essential newborn care.

### Maternal level of knowledge about neonatal danger signs

According to knowledge score of key WHO adopted neonatal danger signs, maternal knowledge has been categorized as good (for scores ≥3) and poor (for scores < 3). Based on the score, more than three-fifth of the mothers 224 (61.7%)] mentioned at least three of the key neonatal danger signs (had good knowledge) whereas 139 (38.3%) mothers mentioned less than three of the signs (had poor knowledge). Among the mothers with poor knowledge of key neonatal danger signs, 28(20.1%) mentioned two danger signs, 98(70.5%) mentioned only one danger sign but the rest 13 (9.4%) mentioned none. From the knowledge score table, the minimum and maximum knowledge scores were 0/9 and 6/9 respectively [Table 8]. The most commonly mentioned neonatal danger signs were fever 130(37.1%), being unable to breastfeed 111(31.7%) and difficulty of breathing 29(8.3%) [Table 9]. The major sources of information for mothers to know the danger signs were health professionals (47.4%), health extension workers (41.7%) and mass media (28.3%).

**Table 8:** Knowledge score of mothers at discharge from NICU, DTGH, North Central Ethiopia, 2019 [*n* = 363]

Knowledge score (out of 9)	Frequency	Percentage	Knowledge category
0	13	3.58	Poor
1	98	27.00	Poor
2	28	7.71	Poor
3	81	22.31	Good
4	69	19.00	Good
5	47	12.95	Good
6	24	6.61	Good

**Table 9:** The key WHO adopted key neonatal danger signs mentioned by mothers at discharge from NICU of DTGH, Debre Tabor town, North Central Ethiopia, 2019

Factor	Percentage
^*^key neonatal danger signs (*n* = 350)	
Fever	37.1
Unable to breastfeed	31.7
Difficulty of breathing	8.3
Lethargy/unconsciousness	6.6
Coldness /hypothermia	4.9
Yellow palms and soles	4.6
Pus discharge from the umbilicus	3.1
Convulsion	2.6
Redness/ eye discharge	1.7

* refers to jaundice, birth trauma

### Determinants of maternal knowledge of neonatal danger signs

Using binary logistic regression model, nine of the total variables were associated with maternal knowledge of neonatal danger signs from bivariable analysis (p<0.25). These were maternal residence, maternal age, maternal level of education, number of antenatal care visits, birth preparedness, place of delivery, spousal accompany during postnatal care, receiving neonatal danger sign information during stay at NICU and knowledge of Essential Newborn Care (ENC). However, from multivariable analysis, only six of these variables were significantly associated with maternal knowledge of neonatal danger signs (*p* < 0.05). These were maternal level of education, number of antenatal visits, birth preparation, place of delivery, receiving danger sign information during stay at NICU and knowledge of ENC.

In this study, mothers with secondary and above educational status were 4.6 times more likely to have good knowledge of neonatal danger signs as compared to mothers who are unable to read and write [AOR = 4.62, 95% CI (1.57, 13.60)]. Moreover, mothers who attended at least 4 ANC visits during their pregnancy were 3 times more likely to have good knowledge about neonatal danger signs as compared to their counterparts (< 4 ANC visits) [AOR = 3.04, 95% CI (1.05, 8.75)]. Institutional delivery was resulted to be a significant factor and hence mothers who gave birth at health institution were 6.5 times more likely to have good knowledge of neonatal danger signs when contrasted to mothers with home delivery [AOR = 6.46, 95% CI (2.74, 15.23)]. Besides, those mothers who were well prepared of their birth were 13.7 times more likely to be aware of neonatal danger signs than those having no well preparation of birth [AOR = 13.70, 95% CI (5.52, 33.98)].

Transfer of information about neonatal danger signs during maternal stay at NICU was of significant in predicting maternal level of knowledge on these signs. Thus, those mothers who received neonatal danger sign information during their several days of stay at NICU were 3.6 times more likely to have good knowledge of danger signs [AOR = 3.64, 95% CI (1.46, 9.07)]. Similarly, mothers who had good knowledge of essential new born care were 4.4 times more likely to know about their neonates’ danger signs as compared to those with poor knowledge of essential new born care [AOR = 4.41, 95% CI (1.76, 11.03)] (Table 10).

**Table 10:** Determinants of maternal level of knowledge about neonatal danger signs among postnatal mothers visiting Neonatal Intensive Care Unit of Debre Tabor General Hospital, Debre Tabor town, North Central Ethiopia, 2019 (*n* = 363)

Factor	Maternal Knowledge of neonatal danger signs		95% CI	
	Good	Poor	Crude OR	Adjusted OR
Maternal age (years) (*n* = 363)				
< 25	48 (13.2%)	59 (16.3%)	1	1
25–34	68 (18.7%)	145 (39.9%)	1.74 (1.08, 2.80)	1.62 (.56, 4.67)
> 34	23 (6.3%)	20 (5.5%)	.71(.35, 1.44)	.54 (.12, 2.46)
Level of education (*n* = 363)				
Unable to read and write	65 (17.9%)	71(19.6%)	1	1
Primary education	46 (12.7%)	77 (21.2%)	1.53 (.93, 2.52)	2.17 (0.72, 6.60)
Secondary and above	28 (7.7%)	76 (20.9%)	2.49 (1.44, 4.30)	4.62 (1.57, 13.60)^*^
Residence (*n* = 363)				
Rural	109(47.2%)	122(52.8%)	1	1
Urban	115(87.1%)	17(12.9%)	7.57(4.28, 13.40)	0.94(.30, 2.91)
Number of ANC visits (*n* = 308)				
< 4	81(39.9%)	122(60.1%)	1	1
≥ 4	88(83.8%)	17(16.2%)	7.80(4.32,14.07)	3.04 (1.05, 8.75)^*^
Birth preparedness (*n* = 363)				
Well prepared	173(90.1%)	19 (9.9%)	20.84 (11.94,37.80)	13.70 (5.52, 33.98)^***^
Not well prepared	52(30.4%)	119(69.6%)	1	1
Delivery place (*n* = 363)				
Health institution	191(84.1%)	36 (15.9%)	16.56 (9.75, 28.12)	6.46 (2.74, 15.23)***
Home	33 (24.3%)	103(75.7%)	1	1
Spousal accompany to PNC(*n* = 363)				
Yes	47 (51.6%)	44 (48.4%)	0.57 (0.35, 0.93)	1.73 (.68, 4.41)
No	177(65.1%)	95 (34.9%)	1	1
Received danger sign information during stay at NICU (*n* = 363)				
Yes	195(84.8%)	35(15.2%)	19.98 (11.57,34.52)	3.64 (1.46, 9.07)**
No	29 (21.8%)	104(78.2%)	1	1
Knowledge of essential newborn care (*n* = 363)				
Good	181(87.0%)	27(13.0%)	17.46 (10.22,29.84)	4.41(1.76, 11.03)**
Poor	43(27.7%)	112(72.3%)	1	1

*Significant at < 0.03, ** significant at ≤0.01 and *** significant at ≤0.001

## Discussion

The study showed that knowledge of neonatal danger signs among postnatal mothers visiting NICU was 61.70%. Having secondary and above level of education, attending more than four ANC visits, birth preparedness, institutional delivery, receiving danger sign information during stay at NICU and good knowledge of essential newborn care were significant determinants for the odds of good maternal knowledge on neonatal danger signs.

In this study, the proportion of mothers with good knowledge of neonatal danger signs was 61.7%. This proportion was lower than the level reported in India 68.9% (15) but higher than findings from North India 50% [20], Nigeria 30% [16], Kenya 15.5% (23), Southwestern Uganda 30.3% [15] and various studies in Ethiopia [6–14]. From this study, the relatively higher level of knowledge may be due to maternal interview after their several days of exposure to NICU environment, where they could learn danger signs. Moreover, methodological differences among the studies and cultural variations among study subjects might have contributed for the differences in level of knowledge.

This study revealed that higher number of antenatal care visits had significant positive association with maternal knowledge of neonatal danger signs and it was congruent with the study conducted in Kenya [15]. The most likely reason could be if a mother attends ANC clinic more frequently, she can be more informed of neonatal danger signs from health care professionals [5]. Therefore, efforts should be maximized to ensure maternal attendance of at least 4 ANC visits in the health care delivery system.

Institutional delivery was found to be associated with increased odds of good maternal knowledge on neonatal danger signs. The possible explanation could be due to the fact that mothers who deliver at health institutions can attend at least the first PNC visit. This helps mothers to be informed of the subsequent PNC appointments during which they can receive neonatal danger signs information [5, 18]. Hence, all personals of the health care system should raise their efforts on awaking mothers to deliver at their nearby health institutions. Moreover, according to studies elsewhere, women’s groups have been effective educational vehicles and can be properly practiced in Ethiopia to increase awareness of institutional delivery and newborn healthcare in the communities [5, 17–19].

Mothers with well preparation of their birth had 13.7 times higher odds of good knowledge on neonatal danger signs. This may be due to the fact that those mothers who were well prepared of their birth often have their ANC visits completed [5]. Furthermore, these mothers are most likely to give their birth at health facility. They are also supposed to be aware of the likely maternal and neonatal complications after birth [5, 18].

Mothers who received danger sign information from neonatal care providers at NICU were 3.64 times more likely to mention at least three neonatal danger signs during discharge interview. Therefore, neonatal care providers should teach mothers regularly about neonatal danger signs by exemplifying their neonates’ real scenario (easier to recall) during their several days of stay at NICU. This in turn helps boost maternal understanding towards the danger signs of their neonatal health so that they can early detect and seek immediate care [5, 17–19].

Mothers who had good knowledge of essential newborn care were 4 times more likely to know neonatal danger signs as compared to those who had poor knowledge of essential new born care. This finding was consistent with a study held at Southern Ethiopia where the odds of knowledge of neonatal danger signs among mothers having good knowledge of essential newborn care was 4.4 times greater than those with poor knowledge of essential newborn care [17]. The consistence may be due to the fact that good knowledge of essential neonatal caring involves maternal understanding of thermal care, breastfeeding, breathing pattern and level of consciousness thereby giving basis for their knowledge of neonatal danger signs.

### Limitation of the study

Findings of this study are supposed to have substantial contribution for the promotion of immediate neonatal health care seeking behavior. This maternal behavior in turn serves like a spring board to achieve the sustainable development goal by reducing Ethiopian neonatal mortality rate to 12/1000 live births by 2030. However, despite our data quality control measures, findings of the study could have been influenced due to interviewer’s bias, maternal socially desirable answers for some factors (E.g. reducing parity which is culturally accepted) and maternal failure to recall of pertinent data like her age. Some important variables such as ethnicity and cultural beliefs were not also considered because asking such issues was sensitive due to the divisive implication, which everyone was afraid of telling oneself confidently. Moreover, the results may not be representative of the entire Ethiopian situation due to a smaller sample size. Being a cross sectional study, its ability to draw any causal inference was limited. Lack of support with qualitative data is also another limitation. Therefore, further follow up research with qualitative support is recommended to understand the relationship between (spousal accompany to PNC, spousal accompany to ANC, information of danger sign during NICU stay) and maternal knowledge of neonatal danger signs.

## Conclusion

The study showed that maternal knowledge of neonatal danger signs wasn’t comparable to their exposure of NICU environment. The odds of good maternal knowledge of these signs was found to be significantly determined by having secondary and above level of education, attending more than four ANC visits, birth preparedness, institutional delivery, receiving danger sign information during stay at NICU and good knowledge of essential newborn care. Therefore, existing efforts should be enhanced to improve number of antenatal visits, institutional delivery rate and postnatal services in the continuum of maternal and neonatal health care system. This approach helps mothers get good opportunity to raise their awareness of danger signs through key messages.

Moreover, information about neonatal danger signs and the circumstances that necessitate newborns to resume visitation of nearby clinic should be passed to the mothers during their several days of stay at NICU and at time of discharge from NICU. This is an opportunity that should never be missed.

## Data Availability

Data will be available upon request from the corresponding author.

## Abbreviations

ANC: Ante natal care
AOR: Adjusted odds ratio
DTGH: Debre Tabor General Hospital
ETB: Ethiopian Birr
NICU: Neonatal Intensive Care Unit
PNA: Perinatal Asphyxia
PNC: Post Natal Care
UNICEF: United Nations Children’s Fund
USD: United States Dollar
WHO: World Health Organization
